# The positive effect of chick embryo and nutrient mixture on bone marrow- derived mesenchymal stem cells from aging rats

**DOI:** 10.1038/s41598-018-25563-w

**Published:** 2018-05-04

**Authors:** Jia Ma, Yanru Guo, Jialei Hu, Yue Pan, Xia Qi, Huaxin Wang, Li Jia

**Affiliations:** 1 College of Laboratory Medicine, Dalian Medical University, Dalian, 116044 Liaoning Province China; 2 Department of Pathology and Forensic Medicine, Dalian Medical University, Dalian, 116044 Liaoning Province China

## Abstract

The aging of many mammalian tissues is associated with loss of functional adult stem cells, especially bone marrow-derived mesenchymal stem cells (BMSCs). This study was aimed to analyze the biological effect of chick embryo (CE) and nutrient mixture (NM) on the BMSCs of aging rats. The aging rat model was established to be induced by D-galactose (500 mg/kg/d) for 90 days. Meanwhile, aging rats were fed with CE and NM in different dose manner by intragastric administration. At the end of the experimental period, serum was collected from rats and used for BMSCs culture. Flow cytometric analysis was used to investigate the BMSCs surface markers. Alizarin Red and oil red O staining were performed to evaluate the multi-lineage differentiation of BMSCs. The results showed that CE plus NM increased the telomere length of BMSCs and promoted BMSCs proliferation. Moreover, CE plus NM administration promoted BMSCs differentiation into osteoblasts and suppressed differentiation into adipocytes. High-throughput sequencing analysis revealed that there were 326 genes were up-regulated and 59 genes were down-regulated in BMSCs of aging rats treated with CE plus NM. In conclusion, CE plus NM supplement had potential to delay aging through the recovery of BMSCs senescence and could be used as a safe effective approach for nutritional therapy of anti-aging.

## Introduction

With a worldwide demographic shift population, aging has become more and more important social and medical problem^1^. Aging is the combined result of physiological and pathological activities involving molecular, cellular, and organ changes^2^. Aging is related to the accumulation of degenerative factors released by senescent cells, such as free radical, protease and reactive oxygen species (ROS), which lead to the increased rate of apoptosis and degeneration^3^. Many diseases are originated from aging and caused degenerative dysregulation of bone marrow-derived mesenchymal stem cells (BMSCs)^4^. Accumulating evidence has demonstrated that age-related decrease of BMSCs quantity is the result of their lifespan decline^5^.

BMSCs are derived from the mesoderm and within bone marrow^6^. BMSCs are multipotential cells that can be induced to differentiate into various cell types, such as osteoblasts, myoblasts, adipocytes and chondrocytes^7^. It is reported that BMSCs facilitate tissue repair via cell replacement from differentiated cells and remodel the microenvironment by releasing chemokine and growth factors^8^. However, studies have shown that the intrinsic properties of BMSCs such as senescence, osteogenic and adipogenic differentiation potential were markedly changed during aging process^9^. These findings provide strong evidence that advanced age is closely associated with abnormalities of BMSCs quantity and function. Therefore, it is necessary to explore a BMSCs therapeutic measure for delaying aging.

In recent years, it has been acknowledged by food scientist and nutritionist that chick embryo eggs are rich in proteins, amino acids, fatty acids, trace elements and other nutrients^10^. Scholars confirmed that chick embryo egg hydrolysates promoted proliferation of mouse marrow cell and increased lifespan^11^. Nutritional supplements emerge as a promising component in nutritional rehabilitation. Nutrient mixture could improve metabolism, enhance immune function, maintain normal physiology and repair tissue damage^12^. In a previous study, Green *et al*. demonstrated that a nutrient mixture, contain amino acids, taurine, trace elements and vitamins were required for the development and survival of mammalian cells^13^. Chothe *et al*. reported that there nutrient elements have an important role in differentiation of BMSCs into osteoblasts and osteocytes, cell proliferation and collagen synthesis^14^. Our previous data showed nutrient mixture supplementation was contributed to promote peripheral blood cells production and marrow nucleated cells proliferation, increase primitive hematopoietic progenitors as well as repair mitochondrial dysfunction in aplastic anemia mice^15,16^. Furthermore, the chick embryo and nutrient mixture has exhibited synergistic role in aging rats by improving immune function, increasing the antioxidant enzyme activity and repairing organs damage. However, the effects of chick embryo and nutrient mixture on recovering BMSCs potential from senescence and then delaying animal aging have not yet been reported.

In the present study, the aging rat model was established to explore the effect of CE and NM (containing various kinds of amino acids, nucleotides, vitamins, trace elements, soybean phospholipid, pentose, niacin, L-carnitine etc.) on BMSCs from aging rats. Our data gave the proof that nutritional support might contribute to delay BMSCs senescence and serve as a therapy of anti-aging.

## Results

### Identification of BMSCs and telomere length in aging rats

**Figure 1:**
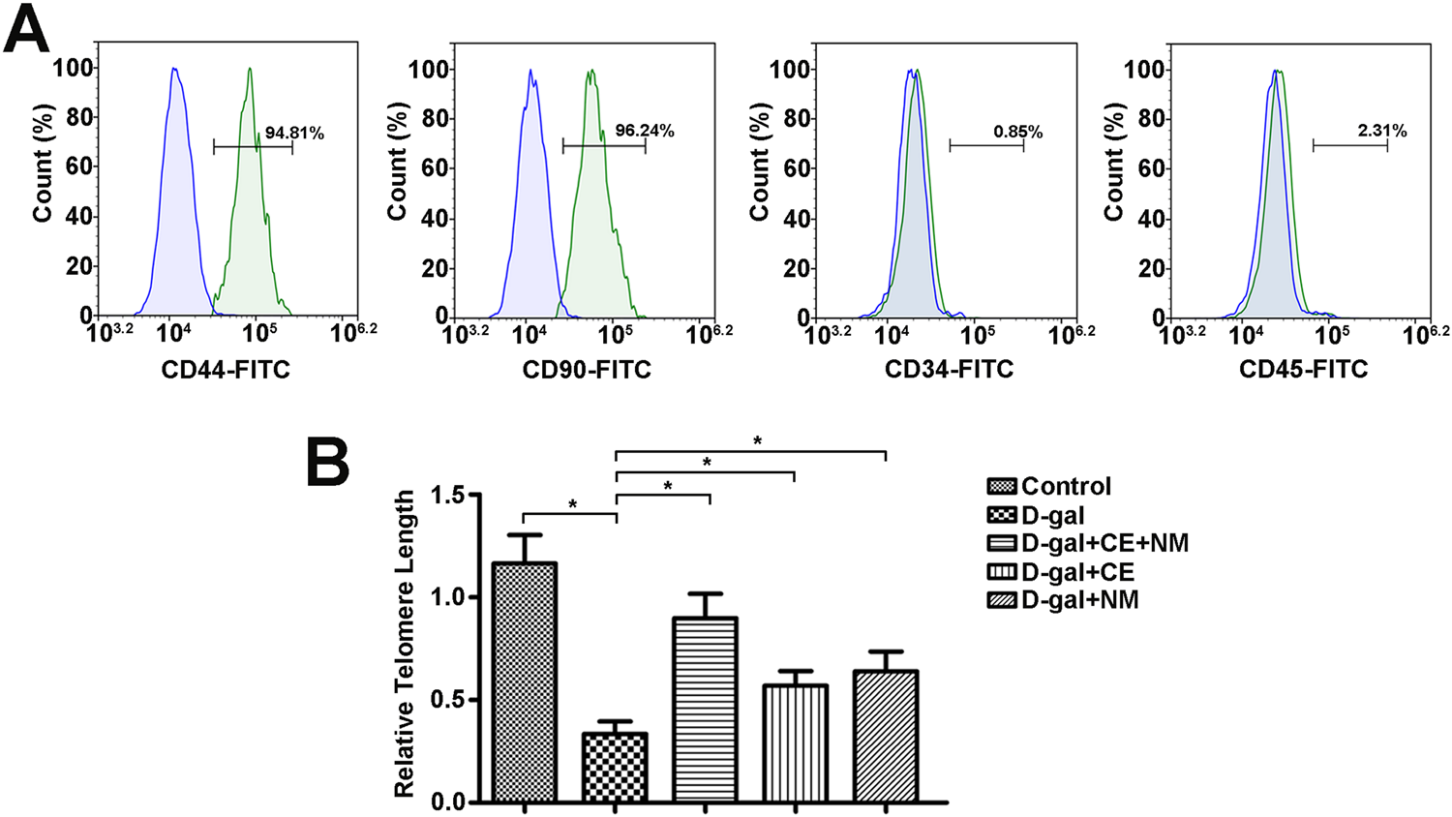
Identification of BMSCs and telomere lengths of BMSCs in aging rats. (**A**) BMSCs were stained with surface markers and examined by flow cytometry. These cells were positive for CD44 and CD90, and negative for CD34 and CD45. (**B**) Real-time PCR technique was used to measure the relative telomere length. The relative telomere length was calculated as the ratio of telomere repeats to a single-copy gene. CE and NM supplement inhibited telomere shortening in aging rat BMSCs. Data were the means ± SD of triplicate determinants (*P < 0.05).

Flow cytometry analysis showed that BMSCs expressed the main positive markers CD44 and CD90. The frequency of CD44 and CD90 positivity was 94.81% and 96.24% in the BMSCs, respectively (Fig. 1A). The main negative markers CD34 and CD45 were expressed in BMSCs only 0.85% and 2.31%, respectively. These results indicated that the cells obtained from rat bone marrow displayed stem cell markers.

Telomere becomes progressively shortened with replication of cells and this feature is widely used to evaluate senescence. The effect of CE and NM on telomere length was evaluated in BMSCs from D-gal-induced rat (Fig. 1B). Expectedly, the relative telomere length was reduced in the D-gal group compared to the control group (*P < 0.05). Compared with D-gal group, the samples in CE and NM treatment groups significantly inhibited telomere shortening (*P < 0.05). Collectively, nutritional supplement of CE plus NM effectively ameliorated telomere shortening.

### CE and NM supplement promote BMSCs proliferation

**Figure 2:**
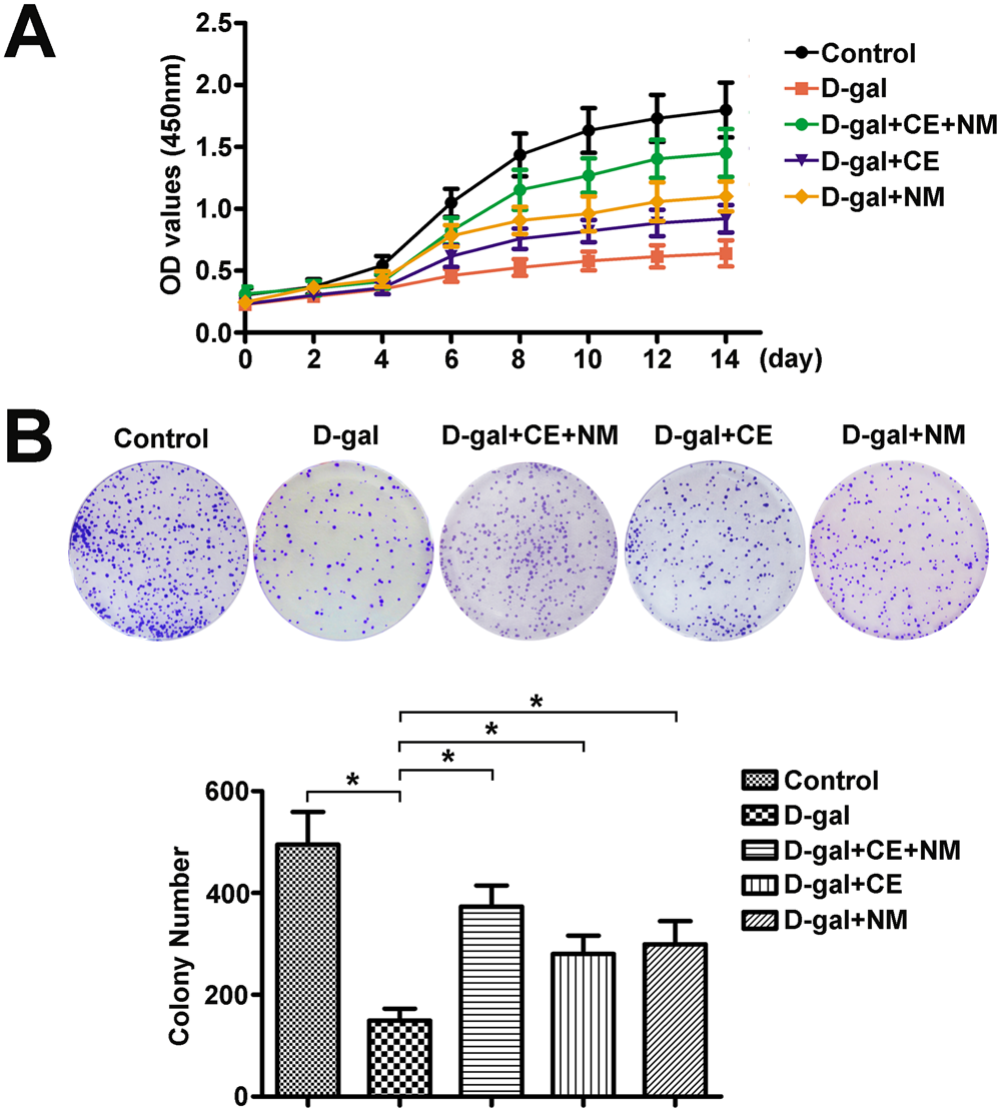
CE and NM supplement effect on proliferation of BMSCs in aging rats. (**A**) BMSCs from different groups were plated in triplicate in 96-well plates at a density of 1 × 10^3^ cells per well, and cell proliferation was examined using CCK-8 kit for 14 consecutive days and growth curve was calculated. (**B**) The colony form of BMSCs was performed in control rats, D-gal rats, CE and NM treated aging rats. Morphology foci were observed at the microscope and photographed. Significant regulation of colony formation numbers was confirmed in BMSCs from D-gal rats compared with control, and CE and NM treated rats compared with D-gal rats. Data were the means ± SD of triplicate determinants (*P < 0.05).

To explore the BMSCs activity among the groups, we performed proliferation assays. According to the results of the CCK8 assay in Fig. 2A, BMSCs started to proliferate in 2–4 days, and the growth continued until the end of the experiment. BMSCs had proliferative potential, although the proliferative capacity was different among the groups. D-gal administration led to significant inhibition of BMSCs proliferation compared to the control group (*P < 0.05). Compared with BMSCs of D-gal-induced rat, the proliferative rate of BMSCs was increased in CE and NM treatment groups (*P < 0.05), while the CE combined with NM group facilitated faster BMSCs proliferation.

To further investigate whether the colony-forming capacity of BMSCs in D-gal rats was associated with CE and NM supplement, the colony formation assay was performed. The colony-forming capacity was decreased in BMSCs of D-gal group compared to the control group (Fig. 2B, *P < 0.05). Interestingly, an increase of colony-forming capacity were observed in D-gal-induced rat treated with CE and NM (*P < 0.05). Thus, CE plus NM supplement could better promote the proliferation of BMSCs in D-gal-induced rat.

### CE and NM supplement enhance the differentiation of BMSCs into osteoblasts in aging rats

**Figure 3:**
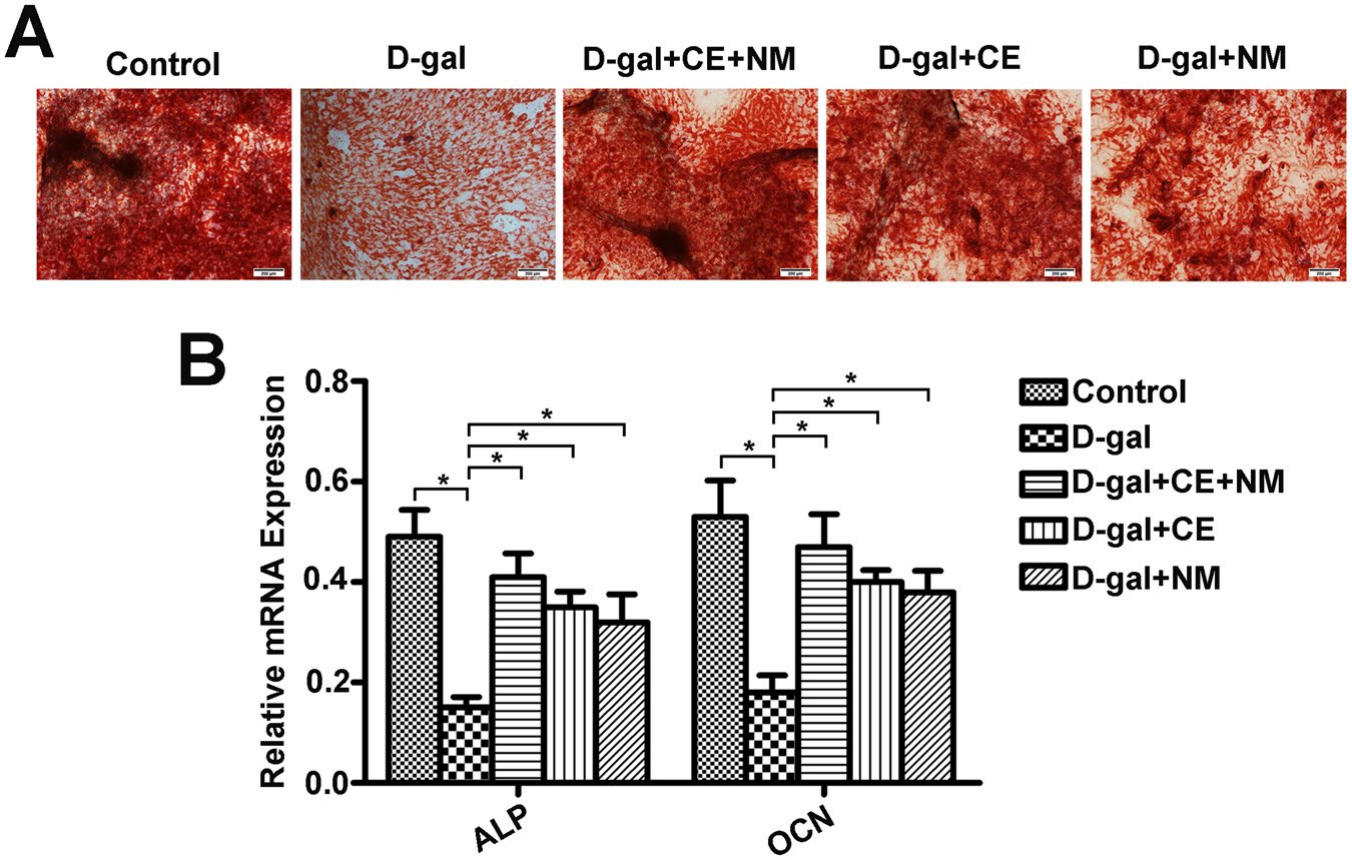
CE and NM supplement mediate the differentiation of BMSCs into osteoblasts in aging rats. (**A**) Alizarine Red staining of aged BMSCs treated with CE and NM after 14 days induction in osteogenic medium was shown. The expression of osteogenic specific genes including ALP and OCN were analyzed by qRT-PCR. The relative expression of each target gene to GAPDH was calculated (*P < 0.05). Values were mean ± SD of three independent experiments.

Above characterization of BMSCs showed significant senescence in D-gal-induced rat. We further determined whether CE and NM treatment could induce BMSCs for osteoblast differentiation. Alizarin Red staining showed that bone matrix mineralization was decreased in D-gal group compared to control group. In contrast, bone matrix mineralization was significantly increased in CE and NM treatment groups (Fig. 3A). To further evaluate the propensity for osteogenic differentiation, the transcription levels of osteoblast specific genes including ALP and OCN were analyzed by qRT-PCR (Fig. 3B). The levels of ALP and OCN mRNAs were considerably decreased in the D-gal group compared with the control group (*P < 0.05). Exposure to CE and NM significantly increased the expression of osteoblast-specific genes compared to D-gal group (*P < 0.05). These results suggested that the presence of CE plus NM markedly inducted osteoblast differentiate of BMSCs.

### CE and NM supplement inhibit the differentiation of BMSCs into adipocytes in aging rats

**Figure 4:**
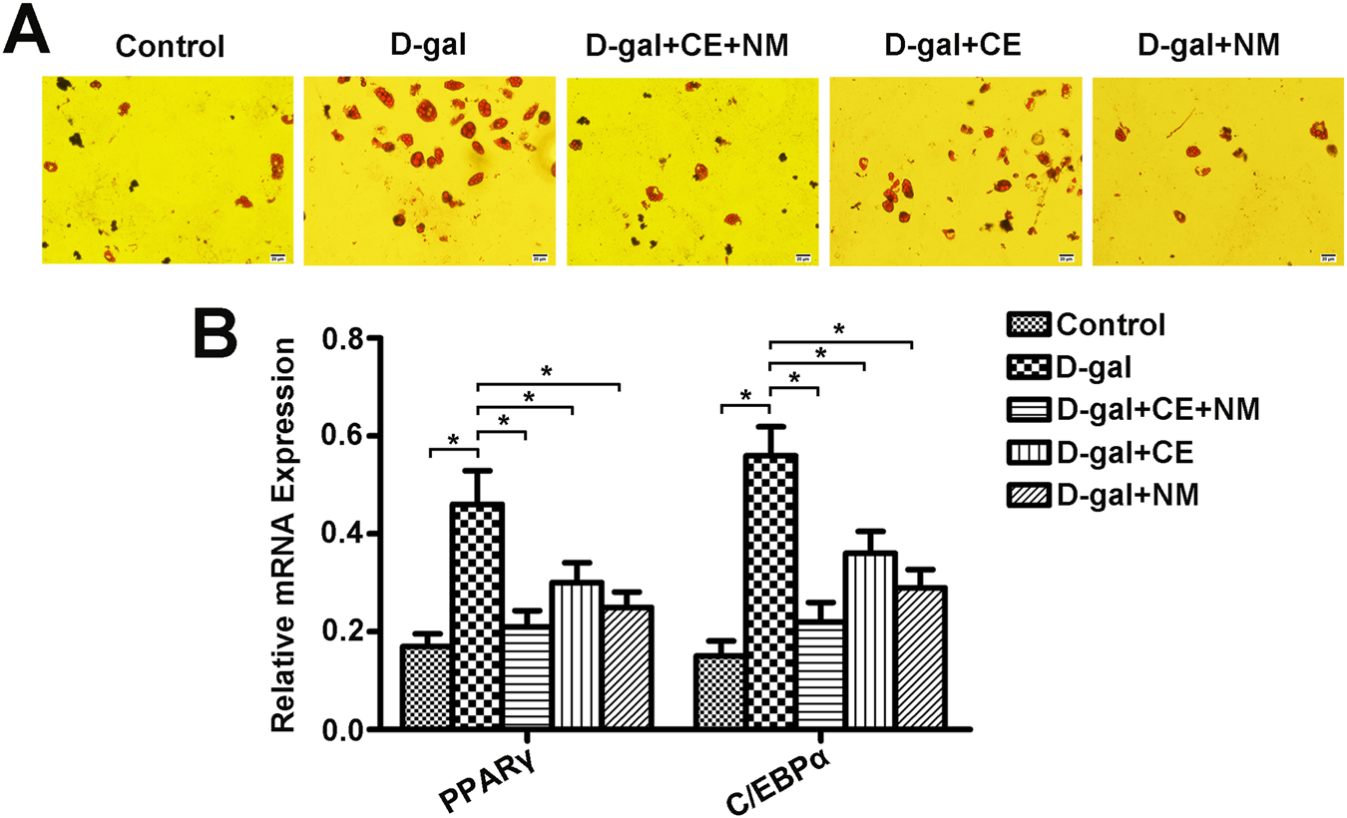
CE and NM supplement effect on the differentiation of BMSCs into adipocytes in aging rats. (**A**) Adipogenic differentiation of BMSCs in aging rats treated with CE and NM for 14 days was detected by Oil Red O staining. (**B**) The levels of PPARγ and C/EBPα mRNA in BMSCs treated with CE and NM were measured by qRT-PCR. The expression of each target gene relative to that of GAPDH was calculated (*P < 0.05). Values were mean ± SD of three independent experiments.

Oil red O staining was applied to evaluate the effects of CE and NM on adipogenic potential of BMSCs. As shown in Fig. 4A, the formation of adipocytes was obviously increased in the D-gal group than that in the control group. Furthermore, a decrease in quantity of red oil drops was showed in the CE and NM treatment groups. Subsequently, the adipocyte-specific genes PPARγ and C/EBPα were analyzed. As shown in Fig. 4B, the levels of PPARγ and C/EBPα mRNAs were up-regulated in the D-gal group compared with the control (*P < 0.05). On the contrary, PPARγ and C/EBPα were down-regulated by CE and NM treatment (*P < 0.05). These data showed that CE and NM played an inhibitory role in adipogenic differentiation of BMSCs, while the effect was more remarkable in CE plus NM treatment group.

### CE and NM supplement mediate gene expression of BMSCs in aging rats

**Figure 5:**
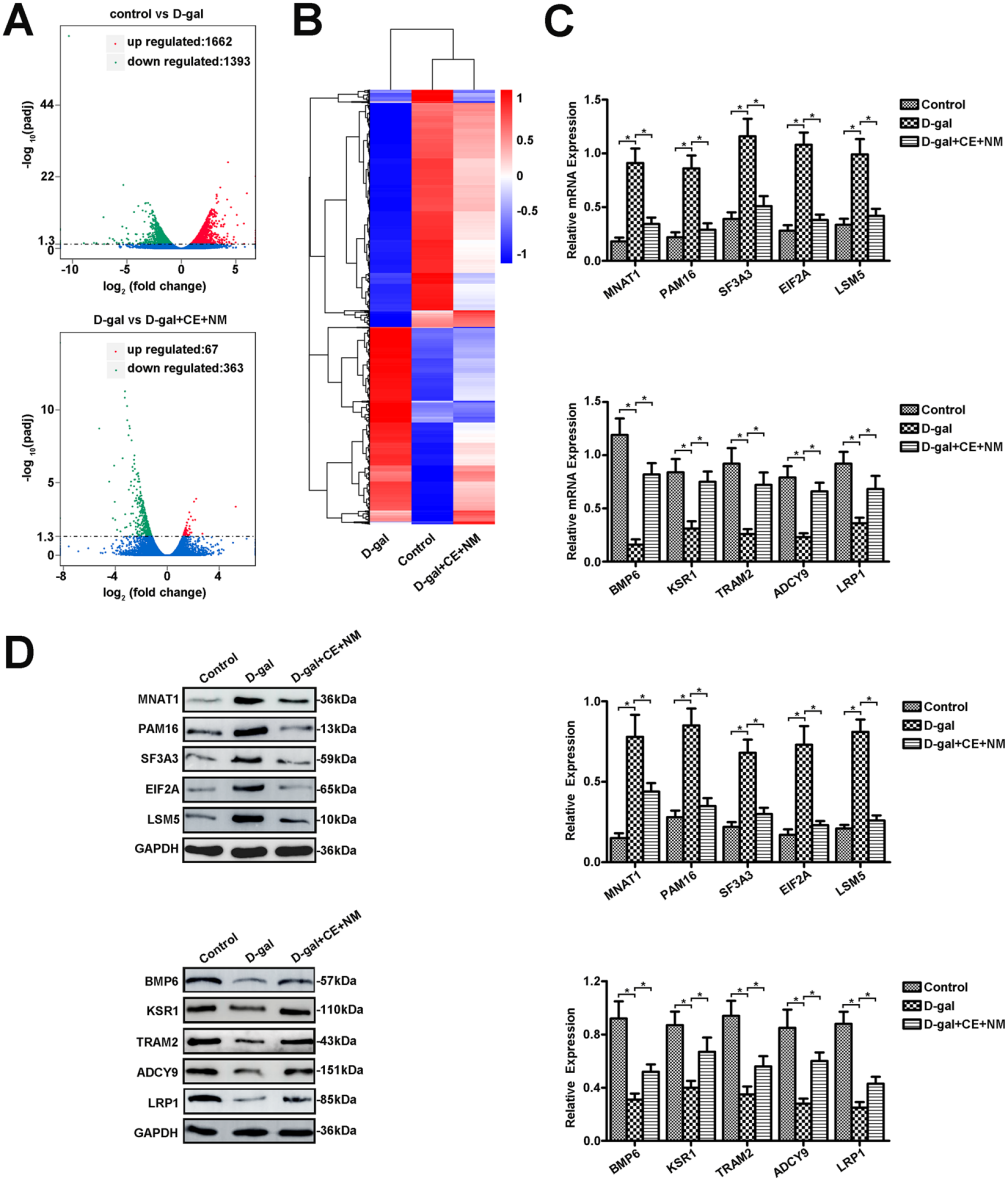
CE and NM supplement mediate the gene expression of BMSCs in aging rats. (**A**) Volcano plot distribution of differential genes was shown. The genes with significantly differential expression were displayed in red dot (up-regulation) and green dot (down-regulation). No significant differential genes were expressed in blue dots. (**B**) Cluster analysis of differential genes was shown. Red showed high expression of genes, and blue showed low expression of genes. The color from red to blue indicated that log10 (FPKM + 1) was from largest to smallest. (**C**) Relative gene levels of up-regulation, including MNAT1, PAM16, SF3A3, EIF2A and LSM5, and down-regulation, including BMP6, KSR1, TRAM2, ADCY9 and LRP1, were determined by qRT-PCR. (**D**) Protein expression was analyzed by Western blot. Values were mean ± SD of three independent experiments.

In order to assess the validity of CE and NM supplement against gene expression in aging rats, the genes of BMSCs were analyzed by high-throughput sequencing analysis. Differential expression of genes was shown by cluster analysis and volcano plot distribution. Volcano plot distribution showed that 1393 genes were up-regulated, while 1662 genes were down-regulated in D-gal group compared with control group (Fig. 5A). In comparison to D-gal group, 363 genes were up-regulated and 67 genes were down-regulated in D-gal + CE + NM group. In addition, cluster analysis of differentially expressed genes showed 59 genes were highly expressed in BMSCs of D-gal group when compared with control group, while those expression in the D-gal + CE + NM group was low (Fig. 5B). In comparison to control group, 326 genes were significantly lower in D-gal group. The 326 genes were increased in D-gal + CE + NM group compared to D-gal group.

Among the 59 genes, 5 genes (>3 folds) were further selected to confirm whether CE plus NM supplement mediated aging progression (Table 1). The mRNA levels of MNAT1, PAM16, SF3A3, EIF2A and LSM5 were up-regulated in D-gal group, while these mRNA were down-regulated in D-gal + CE + NM group (Fig. 5C). Of 326 genes, we selected 5 genes (>5 folds, Table 2). The results showed that the mRNA levels of BMP6, KSR1, TRAM2, ADCY9 and LRP1 were reduced in D-gal group compared to control group. In contrast, these mRNAs were restored in D-gal + CE + NM group.

**Table 1:** The up-regulattion of genes in BMSCs of aging rats.

Associated Gene Name	D-gal vs control	D-gal + CE + NM vs D-gal
Cct8	4.2636825	0.3863017
Chrac1	4.0328507	0.3862749
LSM5	4.0390052	0.3605822
Fnip1	4.1298804	0.2999305
Ifitm3	3.5217683	0.4076209
Ift80	3.1032932	0.2974873
Mfap5	4.4465919	0.3458935
Mnat1	3.123579	0.2556249
Nudt14	4.9293588	0.312299
Pam16	4.357498	0.4196912
Plrg1	4.5287095	0.2836925
Pnrc2	3.6971152	0.3939269
Polr2k	5.969872	0.3742307
Rars	3.5278764	0.3859538
Sf3a3	3.71381	0.3042649
Sucla2	3.5704354	0.4039364
Sugt1	4.5108521	0.3736087
Tax1bp1	3.2636809	0.4026226
Tmem208	4.2486367	0.3504547
Emc6	3.3552029	0.3801129
Ddx3x	3.0831391	0.4143725
Fcer1g	3.1923083	0.3862749
Atp5l	5.335783	0.4192841
Rab9a	4.1118852	0.2401321
Sarnp	5.8138748	0.2135728
Rps27a	3.533995	0.3364817
Hnrnpa1	3.4950183	0.3763899
Ccdc152	3.1579755	0.2743521
Lsm5	7.4024354	0.3225959
Fcgr2b	4.021406	0.3082772
Pfdn4	6.1168971	0.3732205
Zc4h2	3.9413777	0.3077861

Of 59 genes, threefold change of differential genes expression between control and D-gal groups.

**Table 2:** The down-regulation of genes in BMSCs of aging rats.

Associated GeneName	D-gal vs control	D-gal + CE + NM vs D-gal	Associated GeneName	D-gal vs control	D-gal + CE + NM vs D-gal	Associated GeneName	D-gal vs control	D-gal + CE + NM vs D-gal
Ahdc1	0.188979284	3.871788108	Sh3rf3	0.10976576	4.260728135	Flnb	0.161197358	4.400599263
Ankrd63	0.029372299	16.54931961	Spen	0.122792981	3.987542789	Irs2	0.161197358	3.023039857
Atxn1	0.067793934	9.510360309	Ssh1	0.158614877	4.398159729	Hipk2	0.155944005	3.887923952
Bahcc1	0.119302816	5.871788485	Stab1	0.089330708	3.967966924	Igf2r	0.153413782	3.256901301
Bcl9l	0.167368508	4.583659892	Svep1	0.121733657	5.891357086	Pxdn	0.150997859	4.832936368
Bmp6	0.053348814	7.329932942	Synpo	0.190478491	5.141894466	Klf9	0.148085265	4.928333888
Col1a1	0.143547489	4.886494948	Tet3	0.121018541	5.273277797	Ski	0.143995936	4.821559996
Crim1	0.104125837	7.890408538	Tgfbr2	0.158680857	3.647732662	Sulf2	0.141257561	2.540654150
Emc1	0.199091012	2.704946388	Thbs1	0.133424915	4.717426369	Fndc3b	0.139971014	4.145080155
Foxo3	0.124792229	4.557997079	Tlr9	0.140925052	7.271232869	Map1a	0.139004163	5.952517678
Hectd4	0.144676249	3.921756603	Tmem108	0.154577260	4.257185634	Fzd7	0.136853098	4.568751581
Igf1r	0.168275844	4.973632806	Tram2	0.148218764	4.570652066	Soga1	0.134045993	5.596403561
Itgav	0.144275676	4.227486068	Ubr4	0.158900988	3.616514660	Ggn	0.133434164	6.958614086
Ksr1	0.183964589	4.315117966	Zfp827	0.079456001	8.779872565	Col5a1	0.128016351	6.847132830
Lamb2	0.179244406	3.335030603	Srcap	0.196540699	2.81493186	Lrp1	0.124714404	4.719715834
Lamc1	0.151375119	7.39166821	Loxl1	0.194939722	4.698173526	Adcy9	0.123810022	4.905159333
Lgals3bp	0.196486214	2.640479608	Prrc2b	0.194534778	3.693529243	Wdfy3	0.113353434	4.659903245
Loxl2	0.121127639	3.946024737	Itga11	0.193003645	3.733943247	Klf13	0.106882681	9.430929709
Ltbp3	0.158219574	3.596266662	Runx3	0.191989591	3.302594859	Tnrc18	0.104429412	8.531503447
Maf	0.111676691	4.361728588	Nav1	0.191060308	3.521768309	Ahnak2	0.101841678	5.802602089
Man1c1	0.157399188	3.768525096	Fosl2	0.190214614	3.825371867	Nog	0.086683427	16.09677761
Med13l	0.191936368	3.015296767	Prr12	0.188913801	4.145367481	Dync1h1	0.084383223	6.714127808
Mgat3	0.124671188	5.622454184	Arhgap23	0.179916574	3.844510714	Arid1a	0.084219611	7.539662786
Ncor2	0.163504245	4.256005455	Cbarp	0.178253215	3.899529324	Foxd2	0.015438753	37.73941095
Ndst1	0.177206079	3.863745316	Fzd1	0.177673448	3.942470610			
Notch1	0.140700562	3.665220516	Tnrc6c	0.169141197	3.764087069			
Notch2	0.150027617	3.074602705	Npr2	0.166373781	3.057176827			
Otud1	0.121362953	9.661183964	Ldlr	0.162060006	2.760826066			
Runx1	0.169846101	3.312223442	Atn1	0.161208531	6.259728130			

Of 326 genes, fivefold change of differential genes expression between control and D-gal groups.

The protein levels of these ten molecules were further confirmed by western blot. MNAT1, PAM16, SF3A3, EIF2A and LSM5 showed enhanced expression in D-gal group compared to control group, and declined levels in response to CE plus NM treatment (Fig. 5D). BMP6, KSR1, TRAM2, ADCY9 and LRP1 levels were down-regulated in D-gal group compared to control group, while these proteins were up-regulated in D-gal + CE + NM group. In short, CE plus NM supplement mediated gene expression of BMSCs in aging rats.

## Discussion

Increasing studies have demonstrated adult stem cells, including BMSCs, suffered the effect of ageing and reduced their self-renewal and differentiation capacity^17^. The extrinsic cellular factors were indispensable for maintaining BMSCs function^18^. Serum from old mice markedly induced BMSCs dysfunction^19^. Thus, nutritional therapy was valuable in improving bone marrow microenvironment and counteracting BMSCs degeneration to prevent aging. Our current study demonstrated that chick embryo from egg contained different growth factors, such as stem cell factor (SCF), nerve growth factor (NGF), epidermal growth factor (EGF), interleukin-4 (IL-4) and interleukin-2 (IL-2), played crucial role in proliferation, differentiation and survival of different cells^20^. Indeed, taurine was proved having the proliferation promoting and anti-replicative senescence effect on rat BMSCs^21^. Vitamin C supplementation significantly rescued the BMSCs from oxidative stress by regulating autophagy^22^. Moreover, Zn stimulated differentiation and proliferation of osteoblasts by effectively inhibiting osteoclastic and adipocytic differentiation of BMSCs^23^. Halimeh and colleagues demonstrated that L-carnitine supplementation created favorable condition for the growth and survival of rat adipose tissue derived mesenchymal stem cell, and decreased oxidative damage in aging process^24^. Considering that, to better elucidated the effect of functional food on the BMSCs of D-gal-induced aged rat model, we chose to administer CE and NM (52 ingredients, including different amino acids, nucleotides, vitamins, trace elements, soybean phospholipid, pentose, niacin, L-carnitine etc.) in the daily diet of aging rats.

The BMSCs were positive for CD44, CD90 and nearly negative for CD34 and CD45, Which were regarded as the specific markers of BMSCs^25^. In this study, the cultured cells expressed CD44 and CD90 but not CD34 and CD 45, which was in agreement with previous findings. Telomere shortening was now regarded as being a key determinant of an organism’s life expectancy and overall health^26^, and limited BMSCs function during aging. Folate concentration was linked with telomere length and maintained telomere integrity in aging^27^. Farahzadi R *et al*. reported that Zinc sulfate promoted telomere length extension via increasing telomerase gene expression^28^. Furthermore, the dietary antioxidants supported the maintenance of telomere length, particularly vitamin and soybean phospholipid^29^. In the present study, CE plus NM could effect on the relative telomere length of BMSCs.

Factors including age and culture condition affected the proliferation and differentiation of BMSCs^30^. The proliferative rate of BMSCs from young rats was higher than those obtained from elder rats^31^. Jiang *et al*. indicated that Vitamin D remarkably promoted BMSC viability, migration and chondrogenic differentiation^32^. In this work, we developed an aging rat model induced by D-gal, which was now recognized as an inducer of aging reagents that was accelerated senescence in rats^33^. The BMSCs from D-gal-induced rat impaired functional properties in proliferation and colony-forming capacity. Whereas CE plus NM supplement rescued the inhibitory effect of BMSCs growth in D-gal-induced rat, suggesting that proliferative behavior of BMSCs was mediated at least in part by CE plus NM supplement.

It was reported that the biological behavior of BMSCs have age-related changes, namely the osteogenic and adipogenic transdifferentiation. The osteogenic tendency of aging was decreased, while the adipogenic tendency was increased^34^. Wang *et al*. found that melatonin inhibited the adipogenic differentiation of ferric ammonium citrate-treated BMSCs by detecting the expression of adipogenic-specific gene PPARγ and C/EBPα^35^. Farahzadi *et al*. indicated that zinc sulphate induced the osteogenic differentiation of adipose tissue derived mesenchymal stem cells through increasing ALP and PKA activities, cAMP level and expression of ALP, OCN, Runx2 and BMP2^36^. In this study, CE plus NM supplement accelerated osteoblast and inhibited adipocyte differentiation. Correspondingly, CE plus NM supplement promoted the expression of osteogenesis-related markers, while suppressed the adipocyte gene expression. Thus, CE plus NM treatment was potential to recover the differentiation of BMSCs from aging rats.

Aging-related genes affected BMSCs proliferation and differentiation^37^. Histone acetylation modulated gene expression in MSCs, thereby regulating aging^38^. In this study, the differential genes from BMSCs were analyzed. 59 genes were highly expressed and 326 genes were low expressed in BMSCs when compared with D-gal group to healthy control. While 59 genes were low expressed and 326 genes were highly expressed in CE plus NM supplement group compared to D-gal group. The differentially expressed 10 genes were selected to further be verified. PAM16 gene encoded a mitochondrial protein involved in granulocyte-macrophage colony- stimulating factor (GM-CSF) signaling^39^. According to free radical theory of aging, ROS have been proposed as being critical causes of aging, while PAM16 might be important in reactive oxygen species (ROS) homeostasis^40^. Alternative splicing of EIF2A resulted in multiple transcript variants, and affected protein translation and played a deleterious role in aging process^41,42^. BMP6 regulated biological processes including iron homeostasis, fat and bone development^43,44^. LRP1 was involved in several cellular processes and was decreased in aging^45^. Therefore, CE plus NM might be revert aberrant gene expression, extend longevity, and potentially remedy age-related diseases.

In conclusion, CE plus NM supplement played a critical role in regulating the proliferation and differentiation, and mediated aberrant gene expression of BMSCs in aging rats, providing a new view to understand the protecting and nourishing effect of CE plus NM on BMSCs in aging.

## Materials and Methods

### Chick embryo and nutrient mixture

Chick embryo eggs and nutrient mixture was supplied by Dalian Jinfu Biological Technology Development Co., Ltd (Dalian, China). Our previous research showed that the chick embryo contained the most abundant growth factors on the third day of incubation. Considering that, chick embryo on the third day of incubation was selected for further experiments. Nutrient mixture was composed of different amino acids, nucleotides, vitamins, trace elements, soybean phospholipid, pentose, niacin etc. (Table 3).

**Table 3:** Composition, content, and proportion of nutrient mixture.

Sequence number	Composition	Content(g)	Dose proportion (%)	Sequence number	Composition	Content(g)	Dose proportion (%)
1	Lysine	35.35	7	27	Manganese	0.361	0.07
2	Methionine	23.57	4.668	28	Copper	2.431	0.05835
3	Phenylalanine	23.57	4.668	29	Selenium	1.5	0.035
4	Threonine	5.858	1.166	30	Chromium	0.013	0.00116
5	Tryptophan	5.858	1.166	31	Potassium	0.0093	0.00023
6	Arginine	44.21	8.753	32	Calcium	2.929	0.5835
7	Histidine	23.57	4.668	33	Magnesium	10.94	0.8753
8	Glycine	5.858	1.166	34	Inositol	36.44	1.166
9	Aspartic acid	8.838	1.75	35	Soybean phospholipid	1.7676	0.35
10	Leucine	5.858	1.166	36	Vitamin C	23.57	7
11	Isoleucine	5.919	1.166	37	Vitamin B1	0.1464	0.0292
12	Valine	8.838	1.75	38	Vitamin E	0.2947	0.05835
13	Serine	5.919	1.166	39	Glutamic acid	17.676	3.5
14	Glutamine	17.676	3.5	40	Proline	5.858	1.166
15	Taurine	4.42	0.8753	41	γ-aminobutyric acid	2.947	0.5835
16	Orotic acid	5.858	1.166	42	Egg yolk lecithin	22.69	4.085
17	Nucleotide	70.7	14	43	Cephalin	5.858	1.166
18	Vitamin A	0.0236	0.00468	44	Choline	14.645	2.9178
19	Vitamin D	0.0003	0.000058	45	α-linolenic acid	29.29	5.8356
20	Vitamin B2	0.0884	0.0175	46	γ-linolenic acid	14.645	2.9178
21	Vitamin B6	0.0884	0.0175	47	L-carnitine	8.838	1.75
22	Vitamin B12	0.0175	0.000035	48	Pentose	5.858	1.166
23	Niacin	2.929	0.5835	49	Hydroxytyrosol	29.15	1.166
24	Folic acid	0.5858	0.1166	50	Natrium carbonicum	2.653	0.5252
25	Iron	0.0262	0.0052	51	Tyrosine	5.858	1.166
26	Zinc	2.5735	0.0875	52	Cysteine	5.858	1.166

According to the Chinese Nutrition Society in 2013, nutrient mixture was composed of 52 ingredients, including different amino acids, nucleotides, vitamins, trace elements, etc.

### Animal and treatment

Experiments were approved by the Animal Studies Ethics Committee of the Dalian Medical University, China (registered number SYXK 2013–0006). All experiments were performed in accordance with relevant guidelines and regulations. The male SD rats (mean body weight 189.5 ± 21.6 g) were obtained from Animal Facility of Dalian Medical University. 75 rats were randomly divided into five groups with fifteen in each group: Control group, D-gal model group (D-gal) and three D-gal rat groups with chick embryo and nutrient mixture supplement in different doses (chick embryo plus nutrient mixture, chick embryo and nutrient mixture group). Except control group, the other four experimental groups were injected with D-gal (500 mg/kg/day, Sigma, St Louis, MO, USA) for 90 days. The control group was treated with the same volume of physiological saline. The rat of chick embryo group (D-gal + CE) was received daily intragastric administration of 1 ml embryonic chick extract for 90 days. The rat of NM (D-gal + NM) group was fed with NM in a different dose (1–15 days, 0.3816 g/d; 16–30 days, 0.7632 g/d; 30–90 days, 1.1448 g/d) by intragastric administration. The rat of CE plus NM group (D-gal + CE + NM) was received intragastric administration of CE plus NM. The control group and D-gal group were given equal volume of physiologic saline supplemented diet. Besides these extra CE and NM, rats were received a regular diet. After treatment with physiological saline, CE and NM for 90 days, the rats were humanely euthanized and then were determined in the following experiments.

### Isolation of serum

Blood was collected from SD rats in each group by extracting the eyeball blood. Blood was clotted at 37 °C for 4 h. Serum was isolated after centrifugation (10000 rpm for 15 min). Supernatant was collected. Serum from rats was added to the culture of BMSCs in groups.

### Isolation and culture of BMSCs

The femoral bones were separated out from SD rats, soaked in 75% alcohol, and flushed three times in phosphate buffer solution (PBS). Bone marrow cell suspension were prepared by flushing the diaphysis with F-12K and 10% fetal bovine serum (FBS) through syringe needles for three to five times. Bone marrow cells were plated in 75 cm^2^ tissue flasks in 10 ml of BMSCs media at 37 °C with 5% CO_2_. After 72 h, the supernatant and non-adherent cells were removed and fresh medium was added. When BMSCs achieved 85% confluence, adherent cells were trypsinized and collected for further assays. In the present study, the cells from passages 2 to 4 were used for subsequent experiments.

### Telomere length analysis

The relative telomere length was measured using the fluorescence quantitative polymerase chain reaction (PCR). The relative telomere length was calculated as the ratio of telomere repeats to a single-copy gene (SCG) (T/S). The upstream primer sequence and downstream primer sequence of telomere gene was as follows: 5′-CGGTTTGTTTGGGTTTGGGTTTGGGTTTGGGTTTGGGTT-3′ and 5′-GGCTT GCCTTACCCTTACCCTTACCCTTACCCTTACCCT-3′. The upstream primer sequence and downstream primer sequence of the single gene (36B4) was as follows:

5′-AGCGGACCAAACATCCTAACC-3′ and 5′-CATAGGCCCCTGTCACACTCTG-3′. All quantitative (q) PCR were performed on the Thermal Cycler Dice Real time System Single and Lite (TaKaRa, Kyoto, Japan). The cycling profile for the telomere PCR was follows: 95 °C for 10 min, followed by 40 cycles of 95 °C for 15 s and 60 °C for 60 s. Each sample contained three parallel samples, and the average value was taken to calculate T/S.

### Flow cytometry

Surface markers of BMSCs were identified using flow cytometric analysis. Approximately 1 × 10^6^ BMSCs were collected from the passage 2 cultures and washed twice with PBS^46^. After removing the supernatant, the cells were preincubated with 5% BSA for 30 min to block nonspecific binding. The antibodies, including CD44, CD90, CD34 and CD45 conjugated to FITC (BioLegend, USA), were added and incubation for 90 min at 37 °C. After washing with PBS, 500 μl of solution buffer was added to cell pellet and the cells were transferred to flow cytometry tubes. Then, cells were analyzed by a fluorescence-activated cell-sorting (FACS) flow cytometer (BD, Biosciences, CA, USA).

### Cell proliferation

Cell proliferation was measured using cell counting kit-8 (CCK8; KeyGEN, Nanjing, China) according to the manufacturer’s instruction. BMSCs were trypsinized and seeded in 96-well culture plate at the density of 1 × 10^3^ cells per well. The media were replaced every 2 days. Cell proliferation was tested on days 2, 4, 6, 8, 10, 12 and 14 days. 10 μl CCK8 were added to the wells and cultured for an additional 4 h. Then the absorbance was determined at 450 nm using a microplate reader (Bio-Rad Laboratories Inc, Hercules, CA, USA). Each count was an average of three repeats.

### Colony formation assay

Colony formation assay was performed to measure the capacity of cell proliferation. Cells (1 × 10^3^ cells/well) were plated in 6-well plates. The cultures were maintained in the F-12K containing 10% FBS and rat’s serum, with medium changed every 3 days, until the appearance of foci from transformed cells was evident. Cell colonies were fixed with 10% formaldehyde for 40 min, stained with 0.1% crystal violet at room temperature for 20 min, and then photographed.

### Osteoblast differentiation of BMSCs

For osteoblast differentiation, the densities of BMSCs reached 2 × 10^5^ cells/well (passage 4) were plated in 12-well plates. When the culture was at approximately 65–75% confluence, the growth medium was replaced with osteoblast differentiation medium composed of DMEM supplemented with 10% FBS, 10 mM beta glycerophosphate, 50 μg/mL L-ascorbic acid-2-phosphate, 10 nM dexamethasone (Sigma-Aldrich, Denmark) and rat’s serum. The medium was changed every three days during induction period. Alizarin Red staining (Sigma Aldrich™) were performed for the visualization of calcium nodules. Osteoblasts were fixed with 10% paraformaldehyde for 40 min at room temperature, rinsed with PBS, and stained with 0.1% Alizarin Red for 10 min. Images were captured using a digital camera system (Olympus DP12–2) coupled to an inverted optical microscope (Olympus CKX41-Olympus Optical CO., Ltd.; Japan).

### Adipocyte differentiation of BMSCs

The 4 × 10^4^/well cells (passage 4) in 6-well plates were incubation and allowed to reach 65–75% confluent. Adipocyte was induced by the adipogenic differentiation medium composed of 10% FBS, 0.5 mmol/L 1-methyl-3-isobutylxanthine (IBMX), 1 μmol/L dexamethasone, DMEM supplemented with 10 mg/L insulin and rat’s serum. After BMSCs were incubated with adipogenic-induction medium for 14 days, cells were fixed with 10% formaldehyde for 40 min. Followed by washing with PBS and were stained with 0.3% oil red O (Sigma Alrich, St Louis, MO, USA) solution for 30 min at room temperature. Images were captured and observed.

### High-throughput sequencing analysis

Total RNA was extracted from BMSCs with Trizol reagent according to the manufacturer’s instruction. Sequencing library was generated using NEBNext Ultra RNA Library Prep Kit (Illumina, NEB, USA) and index codes were added to attribute sequences to each sample. First strand cDNA was synthesized using random hexamer primer and M-MuLV Reverse Transcriptase (RNase H^−^). Second strand cDNA synthesis was subsequently performed using DNA Polymerase I and RNase H. Remaining overhangs were converted into blunt ends via exonuclease/polymerase activity. PCR products were purified (AMPure XP system) and library quality was assessed on the Agilent Bioanalyzer 2100 system.

### Quantitative real-time PCR (qRT-PCR)

Total RNA was isolated from cultured cell using Trizol reagent, and cDNA was synthesized with QuantiTect Reverse Transcription Kit (QIAGEN, Valencia, CA) according to the manufacturer’s instruction. qRT-PCR was carried out using SYBR-Green-quantitative real-time PCR Master Mix kit (Toyobo Co., Osaka, Japan) and normalized to GAPDH. The relative expression levels of each target gene were determined by using Biosystems 7300 Real-Time PCR system (ABI, Foster City, CA, USA).

### Western blot

Total protein was extracted, separated by 10% SDS polyacrylamide gel electrophoresis and transferred to PVDF membrane (Millipore, Bedford, MA, USA). Membranes were blocked with 5% non-fat dry milk in TBST and were incubated with following primary antibodies: anti-MNAT1 antibody, anti-EIF2A antibody, anti-KSR1 antibody, anti-ADCY9 antibody and anti- kLRP1 antibody (1:1000 Abcam, Cambridge, UK), anti-PAM16 antibody, anti-SF3A3 antibody, anti-LSM5 antibody and anti-TRAM2 antibody (1:800 Abcam, Cambridge, UK), anti-BMP6 antibody (1:400 Abcam, Cambridge, UK), anti-GAPDH antibody (1:2000 Bioworld, Minnesota, USA) at 4 °C overnight. Secondary antibodies were added, and the bands were visualized using the enhanced chemiluminesczence (Amersham Biosciences, Buckinghamshire, UK). GAPDH was used as the loading control.

### Statistical analysis

Each experiment was performed at least in triplicate, and the measurements were performed in three independent experiments. Data are expressed as means ± standard deviation (SD). Student’s t-test was used to compare the means of two groups. P < 0.05 was considered statistically significant. All analyses were performed using SPSS 17.0 statistical packages (SPSS Inc., Chicago, IL).

### Data availability statement

The datasets generated and analyzed during the current study are available from the corresponding author on reasonable request.
